# Intraligamentous Hematoma of the Anterior Cruciate Ligament

**DOI:** 10.1155/2019/9378632

**Published:** 2019-10-21

**Authors:** Yasuaki Tamaki, Daisuke Hamada, Tadashi Mitsuhashi, Tokio Kasai, Takuya Mishiro, Takahiko Tsutsui, Kenichiro Kita, Koichi Sairyo

**Affiliations:** ^1^Department of Orthopedics, Institute of Health Biosciences, The University of Tokushima Graduate School, 3-18-15 Kuramoto, Tokushima 770-853, Japan; ^2^Department of Orthopedics, Takamatsu Red Cross Hospital, 4-1-3 Ban-cho Takamatsu 760-0017, Japan

## Abstract

Lesions of the anterior cruciate ligament (ACL) are rare entities in clinical practice. Here, we present the case of an intraligamentous hematoma of the ACL. A 20-year-old man (height 173 cm, weight 62.9 kg, body mass index 21) with no significant past medical history developed progressively worsening pain and limitation of range of motion in the left knee due to minor trauma. No abnormality was found on plain radiography; however, magnetic resonance imaging revealed a cystic lesion in the intercondylar fossa that was hypointense on T1-weighted imaging and hyperintense on T2-weighted imaging. We performed knee arthroscopy, made a longitudinal incision in the anterior aspect of the ACL, and identified a hematoma. The patient's postoperative course was uneventful. There is no evidence of recurrence at one year after surgery. Although the ACL is a relatively hypovascular structure, it does contain microscopic blood vessels. In this case, we speculate that the intraligamentous hematoma was the result of rupture of these very small blood vessels in response to a minor injury.

## 1. Introduction

Lesions of the anterior cruciate ligament (ACL) are rarely encountered in clinical practice. There are several reports on ganglion cyst of the ACL and intraligamentous synovial chondromatosis of the ACL [1–4]. The incidence of intraligamentous cyst of the ACL based on magnetic resonance imaging was reported 0.26% to 0.44% [1, 4, 5]. Only one report described the intraligamentous synovial chondromatosis of the ACL [2]. Because the presence of these cystic lesions caused nonspecific symptom of the knee including pain, limited range of motion, mechanical locking, clicking, and swelling, physicians should consider this as one of the differential diagnoses of the symptomatic knee [3–5]. As one of the cystic lesions of the ACL, the intraligamentous hematoma was not described in previous literatures. We described a case of intraligamentous hematoma of the ACL.

The patient was informed that data concerning the case would be submitted for publication, and he provided consent.

## 2. Case Presentation

A 20-year-old man (height 173 cm, weight 62.9 kg, body mass index 21) with no relevant past medical history presented with a complaint of progressively worsening pain in his left knee and limitation of range of motion. Two weeks earlier, he had fallen into a ditch and subsequently developed slight pain in the left popliteal region. He visited our hospital 2 weeks after the injury complaining of sudden pain on deep flexion and limited range of motion of the left knee.

On physical examination, there was no swelling, redness, local warmth, or patellar ballottement in the left knee. Extension of the left knee was limited to -10° and accompanied by pain. Flexion was limited to 95°. A more detailed physical assessment such as the McMurray test, Lachman test, and pivot shift test was not performed because of severe knee pain. The preoperative Knee Injury and Osteoarthritis Outcome Score (KOOS) was 14.1.

**Figure 1:**
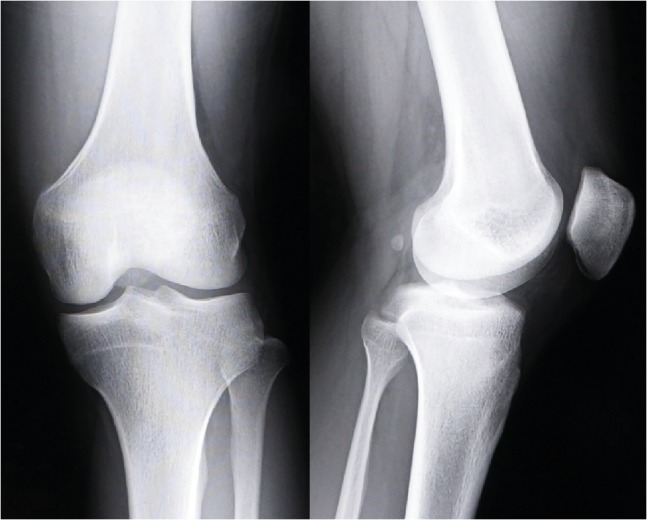
Plain radiograph obtained preoperatively showing no abnormalities.

**Figure 2:**
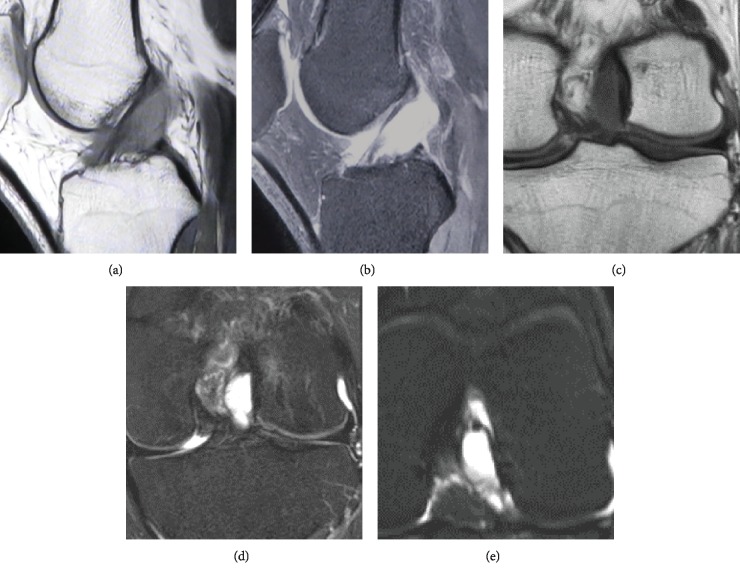
Magnetic resonance images obtained preoperatively. MRI revealed thinning of the ACL and a cystic lesion (*φ*23 × 12 mm) in the intercondylar fossa that was hypointense on T1-weighted imaging and hyperintense on T2-weighted imaging. (a) Sagittal T1-weighted scan. (b) Sagittal T2-weighted scan. (c) Coronal T1-weighted scan. (d) Coronal T2-weighted scan. (e) Axial T2-weighted scan.

No abnormality was found on plain radiography (Figure 1); however, magnetic resonance imaging (MRI) revealed thinning of the ACL and a cystic lesion (*φ*23 × 12 mm) within the ACL that was hypointense on T1-weighted imaging and hyperintense on T2-weighted imaging (Figure 2). No other abnormal findings, such as hemarthrosis, hydrarthrosis, meniscus injury, or cartilage damage, were detected on MRI. The differential diagnosis at this stage included periligamentous or intraligamentous ganglion of the ACL, intraligamentous hematoma of the ACL, and intraligamentous tumor, such as synovial chondromatosis. We performed knee arthroscopy in order to debride the cystic lesion and make a definitive diagnosis.

**Figure 3:**
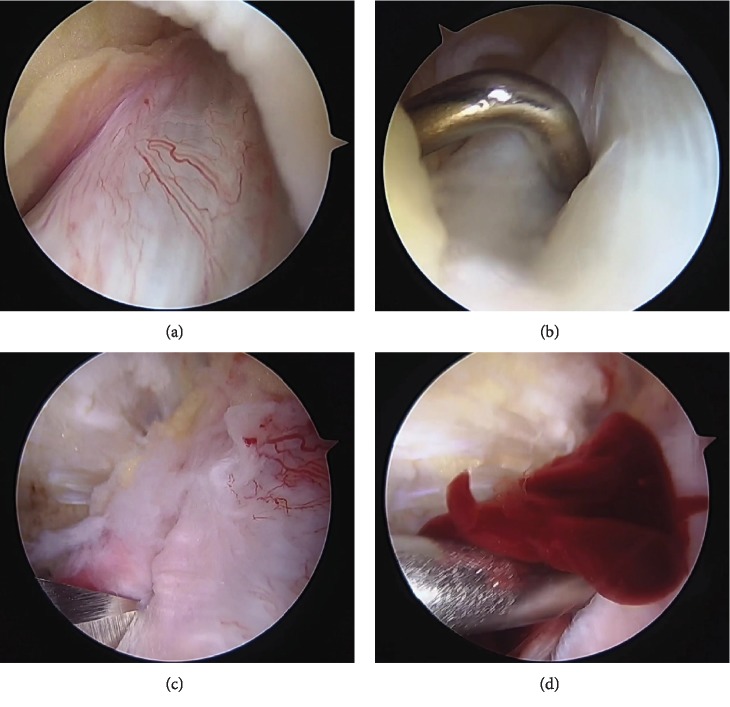
Findings on arthroscopy. (a) The anterior cruciate ligament (ACL) is slightly swollen but intact. (b) There is no cystic lesion around the ACL. (c) Longitudinal incision made on the anterior aspect of the ACL. (d) Discharge from an intraligamentous hematoma.

There were no abnormal arthroscopic findings in the articular cartilage or meniscus (Figure 3). The ACL was slightly swollen but otherwise intact with good tension. No cystic lesion was detected in the vicinity of the ACL. We then made a longitudinal incision on the anterior aspect of the ACL, which revealed the hematoma. The lesion did not include a viscous liquid-like ganglion or any solid tissue such as a chondral body. We only performed removal of the hematoma using an arthroscopic probe. We did not use any radiofrequency devices because we could not detect the active bleeding around the hematoma.

**Figure 4:**
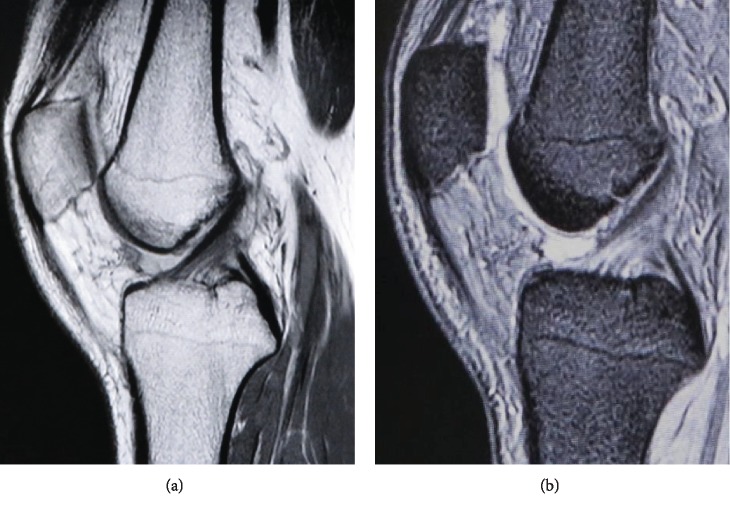
Sagittal (a) T1-weighted and (b) T2-weighted magnetic resonance images acquired one year after surgery confirm complete resolution of the intraligamentous hematoma and an intact anterior cruciate ligament.

The limitation of knee motion was resolved on the day after surgery. Extension of the left knee was 0° and flexion was 140°. There was no pain, swelling, or instability of the left knee during the postoperative period. The patient was discharged from the hospital on postoperative day 6 with a postoperative KOOS of 98.2. Follow-up MRI performed one month postoperatively confirmed no recurrence of the hematoma (Figure 4). The patient has had no symptoms in the left knee in the 12 months since his surgery. MRI performed 12 months postoperatively also confirmed no recurrence of the hematoma.

## 3. Discussion

To our knowledge, this is the first report describing the intraligamentous hematoma of the ACL. Previous reports on lesions of the ACL have described a ganglion cyst or synovial chondromatosis. A ganglion cyst of the cruciate ligament is a rare entity, and most cysts involving ligaments of the knee joint are located in the ACL [1]. MRI findings for a ganglion cyst include low signal intensity on T1-weighted imaging and high signal intensity on T2-weighted imaging. Synovial chondromatosis of the ACL is extremely rare, and the MRI findings include thickening of the ACL and an intraligamentous mass that is hypointense on T1-weighted images and hyperintense on T2-weighted images [2].

In our patient, MRI revealed thickening of the ACL and a cystic lesion adjacent to the ACL that was hypointense on T1-weighted images and hyperintense on T2-weighted images. These findings are similar to those for a ganglion cyst or synovial chondromatosis. Therefore, it is very difficult to distinguish a ganglion cyst, synovial chondromatosis, and intraligamentous hematoma from preoperative MRI findings. Arthroscopy is essential for the definitive diagnosis.

There is general agreement that the ACL is a relatively hypovascular structure. However, there have been some human cadaveric studies of vascularization of the ACL. Toy et al. reported that the terminal branches of the inferior genicular arteries supply the distal portion of the ACL directly [6]. Duthon et al. also investigated vascularization of the ACL and found that the blood supply of the cruciate ligament is provided by the middle genicular artery [7]. Therefore, it is clear that blood vessels exist within the ACL, albeit not with a homogeneous distribution. Although the ACL is a relatively hypovascular structure, it does contain microscopic blood vessels. We speculate that these small blood vessels ruptured as a result of minor trauma, discharging their contents into the ACL and creating an intraligamentous hematoma.

## 4. Conclusion

To our knowledge, this is the first report of intraligamentous hematoma of the ACL. The findings on arthroscopy are essential for a definitive diagnosis. Arthroscopic debridement of the intraligamentous hematoma is effective.
